# *Plasmodium ovale *infection in Malaysia: first imported case

**DOI:** 10.1186/1475-2875-9-272

**Published:** 2010-10-08

**Authors:** Yvonne AL Lim, Rohela Mahmud, Ching Hoong Chew, Thiruventhiran T, Kek Heng Chua

**Affiliations:** 1 Department of Parasitology, Faculty of Medicine, University of Malaya, 50603 Kuala Lumpur, Malaysia; 2 Department of Molecular Medicine, Faculty of Medicine, University of Malaya, 50603 Kuala Lumpur, Malaysia; 3 Sunway Medical Centre Berhad, No. 5, Jalan Lagoon Selatan, Bandar Sunway, 46150 Petaling Jaya, Selangor, Malaysia

## Abstract

**Background:**

*Plasmodium ovale *infection is rarely reported in Malaysia. This is the first imported case of *P. ovale *infection in Malaysia which was initially misdiagnosed as *Plasmodium vivax*.

**Methods:**

Peripheral blood sample was first examined by Giemsa-stained microscopy examination and further confirmed using a patented in-house multiplex PCR followed by sequencing.

**Results and Discussion:**

Initial results from peripheral blood smear examination diagnosed *P. vivax *infection. However further analysis using a patented in-house multiplex PCR followed by sequencing confirmed the presence of *P. ovale*. Given that *Anopheles maculatus *and *Anopheles dirus*, vectors of *P. ovale *are found in Malaysia, this finding has significant implication on Malaysia's public health sector.

**Conclusions:**

The current finding should serve as an alert to epidemiologists, clinicians and laboratory technicians in the possibility of finding *P. ovale *in Malaysia. *P. ovale *should be considered in the differential diagnosis of imported malaria cases in Malaysia due to the exponential increase in the number of visitors from *P. ovale *endemic regions and the long latent period of *P. ovale*. It is also timely that conventional diagnosis of malaria via microscopy should be coupled with more advanced molecular tools for effective diagnosis.

## Background

Malaria is one of the most deadly parasitic diseases in the world, with an annual infection rate of 500 million cases and more than one million deaths. In Africa, malaria remains the single largest killer among children younger than 5 years of age with a mortality rate of 3,000 children per day. The latest malaria statistics recorded 243 million cases with 863, 000 deaths globally [1]. Currently, malaria can be caused by five *Plasmodium *species which include *Plasmodium falciparum Plasmodium vivax, Plasmodium ovale, Plasmodium malariae *and more recently *Plasmodium knowlesi*. Infections caused by *P. falciparum *and *P. knowlesi *may be fatal while the other species generally cause milder disease. Distribution of *P. falciparum *and *P. vivax *are extensively distributed in the tropics and temperate regions of the world. *P. malariae *is also found in these regions but is less common while cases of *P. knowlesi *in on the rise in Southeast Asia [2-5] and *P. ovale *is mainly restricted to sub-Saharan Africa [6].

In Malaysia, malaria remains the most common vector-borne parasitic disease. Although cases have decline in general, malaria is still a public health problem in Sabah and Sarawak. However, more recently, urban malaria cases have surged. Malaria is still clinically significant due to high mortality rates caused by *P. falciparum *in cerebral malaria cases. The recent cases of *P. knowlesi *infections in humans was first brought into international attention in 2004, when Singh *et al *, a Malaysian researcher, highlighted in The Lancet the occurrence of naturally-acquired *P. knowlesi *infections from macaques in human beings when they examined 208 people with malaria in the Kapit division [7]. This created great excitement in the field of malaria research and with an avalanche of information, *P. knowlesi *was subsequently labelled 'the fifth *Plasmodium *species that infects humans'. In the same study, two cases of *P. ovale *were also detected by nested PCR assays. Since then, no other known cases of *P. ovale *infection has been reported. Here, a case of imported *P. ovale *infection confirmed by multiplex PCR targeting the small subunit ribosomal RNA (SSU rRNA) gene is reported. The gene was sequenced and compared with other reference *P. ovale *isolates.

## Case presentation

A 20-year-old Nigerian male student who has been in Malaysia for the last six months presented with a history of fever associated with chills and rigors for the last four days. Other physical examination findings were unremarkable. He was diagnosed as pyrexia of unknown origin (PUO). Laboratory findings on admission revealed anaemia (haemoglobin of 10.8 g/dL), platelet count of 117,000/ml and eosinophils of 2%. Blood examination of the thin smear stained with Giemsa showed enlarged infected red blood cells (RBCs) with putative parasites resembling *P. vivax*. He was treated with quinine 600 mg tds and doxycycline 100 mg bd. One week after admission, the patient was well.

## Confirmation of *Plasmodium ovale *using in-house multiplex polymerase chain reaction (PCR) and sequencing

**Figure 1:**
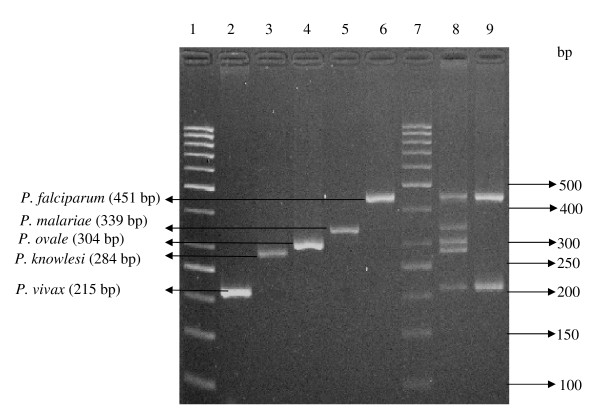
**Multiplex PCR results on *Plasmodium *spp**. Lane 1, 7: 50 bp molecular weight ladder; Lane 2: Patient sample containing *P. vivax*; Lane 3: Patient sample containing *P. knowlesi*; Lane 4: Patient sample containing *P. ovale*; Lane 5: Patient sample containing *P. malariae*; Lane 6: Patient sample containing *P. falciparum*; lane 8: Positive controls with all the bands amplified from 5 types of *Plasmodium *constructs. Lane 9: Patient with mix infection, sample containing *P. falciparum *and *P. vivax*.

Total genomic of this patient's blood DNA together with the *Plasmodium *DNA from the patient was extracted using QIAamp DNA Mini Kit (Qiagen, USA) according to the manufacturer's instructions. The extracted DNA was subjected to a patented in-house PCR targeting the SSU rRNA gene. Following that, the PCR products were electrophoresed on 3% (w/v) ethidium bromide stained agarose gel. Results showed that the patient was infected with *P. ovale *instead of *P. vivax*, as previously diagnosed by conventional microscopy (Figure 1, lane 4). Further confirmation with PCR amplification was also carried out using genus specific primers (rPLU5/rPLU6) followed by sequencing.

## Analysis of sequences and phylogenetic analyses of sequence data

Sequencing data obtained was analysed using BLAST program [8]. The BLAST result showed that the sequence information obtained has 99% similarity to SSU rRNA gene of *P. ovale *in the GenBank. For phylogenetic analyses, the sequence determined were compared with those available in GenBank and published in quality, peer-reviewed scientific journals. Sequences were trimmed and then aligned using the program Clustal × [9], and the alignments were adjusted by employing the program BioEdit [10].

The phylogenetic analysis of the sequence data was conducted using Bayesian inference (BI) and by employing the software package MrBayes v3.1.2. Posterior probabilities (pp) were calculated via 2,000,000 generations (ngen = 2,000,000; burnin = 20,000) using the Monte Carlo Markov chain method, which utilizes four simultaneous tree-building chains (nchains = 4) with every 100^th ^tree saved (samplefreq = 100). The evolutionary distance was calculated using the general time reversible evolutionary model (nset = 6), which allows for a gamma-shaped variation in mutation rates between codons (rates = gamma).

**Figure 2:**
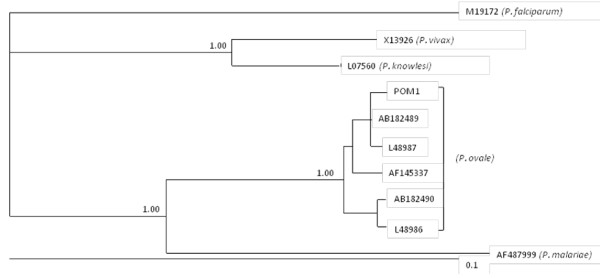
**Phylogenetic analyses of SSU rRNA sequence data representing *Plasmodium ovale *from a patient (POM1) using Bayesian inference (BI)**. Sequences from the present study as well as reference sequences representing *P. ovale, P. falciparum, P. vivax *and *P. malariae *(acquired from GenBank) are indicated. Posterior probabilities are indicated at all major nodes.

*Plasmodium falciparum *[GenBank accession number M19172] [11], *P. vivax *[GenBank accession number X13926] [12], *P. malariae *[GenBank accession number AF487999] [13], *P. knowlesi *[GenBank accession number L07560] [14], were used as the out groups in the analysis of SSU rRNA sequence data for *P. ovale*. Upon the completion of the Bayesian analysis, a 50% majority-rule consensus tree for each species was constructed in Treeview × v.0.5. Analysis of SSU rRNA sequence data inferred the patient was indeed infected with *P. ovale *and not *P. vivax *as initially diagnosed by microscopy (Figure 2).

## Discussion

This is the first case of imported malarial infection caused by *P. ovale *in Malaysia. *P. ovale *is a cause of benign and relapsing tertian malaria and the least common among the human-infecting *Plasmodium *species. Besides *P. vivax, P. ovale *also has a dormant liver stage (with hypnozoites) following primary infection. Mature schizonts develop from the hypnozoites which later release merozoites into the blood stream causing clinical symptoms of malaria (relapsing malaria) even after many months of primary infection. However, the therapy of *P. ovale *infection is similar to that of malaria caused by *P. vivax*.

Globally, *P. ovale *infection causes 0.5-10.5% of all malaria cases. Although the distribution of *P. ovale *is concentrated in sub-Saharan Africa [6], *P. ovale *infections have also been intermittently reported in other parts of the world such as India [15-18], Papua New Guinea [19], Korea [20], Thailand [21], Spain [22] and Sri Lanka [23]. In Southeast Asia, prevalence of *P. ovale *infections ranged from 2.0% to 9.4%, with records from southern Vietnam, Thai-Myanmar border, Lao PDR and Indonesia [24-26].

Although clinically, the treatment for *P. ovale *is the same as *P. vivax*, the specific identification of *P. ovale *is of significant public health importance. Based on enzyme-linked immunosorbent assay-based detection of infected mosquitoes, *Anopheles gambiae *and *Anopheles funestus *are the likely natural vectors of *P. ovale *in Africa [27]. *Anopheles atroparvus *has also been experimentally shown to be an effective mosquito host and capable of transmitting the infection to humans [28-32]. Other proven experimental hosts are *Anopheles albimanus *[33-35], *Anopheles quadrimaculatus *[33,34], *Anopheles freeborni *[36], *Anopheles maculates *[36], *Anopheles subpictus *[32], *Anopheles. stephensi *and *Anopheles balabacensis balabacensis *(= *Anopheles. dirus*) [37]. In studies with the Donaldson strain of *P. ovale*, *A. quadrimaculatus *was the most susceptible to infection, followed by *A. albimanus *from the Florida Keys and *A. albimanus *from Panama [33]. In comparative studies with the West African strain, *A. stephensi *was the most susceptible, followed by *A. freeborni*, *A. dirus*, *A. quadrimaculatus*, *A. maculatus*, and *A. albimanus *[37]. *Anopheles farauti *has also been experimentally infected with *P. ovale *[38]. From these proven experimental hosts of *P. ovale*, two are found in Malaysia. The presence of *A. maculatus *and *A. dirus *in Malaysia increase the public health risk of naturally acquiring the infection. Infected *P. ovale *human host may be bitten by these vectors and the disease can be spread via the bite of the infected mosquito.

While most *P. ovale *infections in Asia were imported cases, there were at least two reports which indicated indigenous cases such as the one in India [18] and the other in Sri Lanka [23]. Since in both cases, patients involved had no travel history or receipt of blood transfusion or any blood products, *P. ovale *infection was postulated as locally acquired through infected mosquito bites [18,23]. These incidences highlighted two possibilities. Firstly, *P. ovale *parasites could have been prevalent in these countries but have never been detected due to high similarities between the morphology of *P. ovale *and *P. vivax *leading to misdiagnosis via conventional microscopy. Secondly, *P. ovale *could have first been introduced into these countries from human host who had been to Africa, where *P. ovale *is endemic. With the presence of suitable mosquito vectors in India and Sri Lanka, *P. ovale *could have been transmitted to other susceptible hosts. However, both articles did not mention about the vectors that were available in each respective country.

Misdiagnosis of *P.vivax/P.ovale *infection is possible because using routine microscopy it is difficult to morphologically distinguish *P. vivax *and *P. ovale *parasites. Both have the characteristic enlarged erythrocytes which are sometimes not obvious on thick/thin blood smears that are routinely used to detect malaria infections. There is also no difference in clinical presentation between infections with *P. vivax *and *P. ovale*, with both parasites resulting in pathognomonic chills and rigors every 48 hours.

Therefore, more advanced and reliable diagnostic techniques, such as PCR, should be utilized to provide specific identification of species, as was the case in Sri Lanka [23]. Conventionally, to identify *Plasmodium *species, microscopic examination of Giemsa-stained blood smears has been the diagnostic method of choice [39]. However, it is not easy to identify *P. ovale *on a blood smear, particularly when parasite numbers are low and mixed species infections are present [40]. The frequency of single *P. ovale *infection is very low even in *P. ovale-*endemic areas, while the frequency of mixed species infection involving *P. ovale *was very high. In these areas, *P. ovale *was more frequently observed using PCR methods (3.8-16.5%) than with conventional blood smear methods (0-0.4%) [24,26,41,42]. A combined application of nucleic acid detection methods with the conventional blood smear methods should be the future recommended method especially for the identification of *Plasmodium *species in mixed species infections and in imported malaria cases.

This is important since there have been several recent reports of *P. ovale *infections in areas of low transmission of malaria in southern and southeast Asia [17,24-26,43]. It is believed that these parasites were introduced into these countries due to increased travel of tourists to or from Africa. With Malaysia's open policies in encouraging foreign workers in construction, agricultural and service sectors, besides providing lots of higher education opportunities at relatively lower cost compared to USA and UK, many foreigners including those from Africa are flocking into the country. Hence, it is paramount that clinicians and diagnostic laboratory technicians are fully aware of the possibilities of novel pathogens being introduced into the country.

This present finding essentially also highlighted the need to consider even historically distant exposure to *P. ovale*, given the potentially long latency period to relapse [44-46]. It is vital to note that an initially negative single malaria smear does not rule out *P. ovale *infection, particularly given its typical low parasitemia [6,46].

## Conclusions

This current finding should serve as an alert to epidemiologists, clinicians, laboratory technicians and other key stakeholders in the possibility of finding *P. ovale *in Malaysia. *P. ovale *should be considered as an etiology for imported malaria in Malaysia. There is a potential risk to public health since the mosquito vectors for *P. ovale *are found in Malaysia. In addition, it is also recommended that there is an urgent need to couple microscopic technique with advanced molecular methods (i.e., multiplex PCR) for accurate and specific diagnosis.

## Competing interests

The authors declare that they have no competing interests.

## Authors' contributions

RM and TT carried out the clinical and laboratory diagnosis. CHC carried out the molecular genetic studies. YALL participated in the sequence alignment and drafted the manuscript. KHC and YALL conceived of the study, and participated in its design and coordination and drafted the manuscript. All authors read and approved the final manuscript.

## Consent

The study protocol (Reference Number: 709.2) was approved by the Ethics Committee of the University Malaya Medical Centre (UMMC), Malaysia before the commencement of the study. Written informed consent was obtained from the patient for publication of this case report.
